# Phenotypic and Functional Changes in Blood Monocytes Following Adherence to Endothelium

**DOI:** 10.1371/journal.pone.0037091

**Published:** 2012-05-15

**Authors:** Colin Tso, Kerry-Anne Rye, Philip Barter

**Affiliations:** 1 Lipid Research Group, The Heart Research Institute, Newtown, New South Wales, Australia; 2 Department of Medicine, University of Sydney, Sydney, New South Wales, Australia; 3 Department of Medicine, University of Melbourne, Melbourne, Victoria, Australia; Bristol Heart Institute, University of Bristol, United Kingdom

## Abstract

**Objective:**

Blood monocytes are known to express endothelial-like genes during co-culture with endothelium. In this study, the time-dependent change in the phenotype pattern of primary blood monocytes after adhering to endothelium is reported using a novel HLA-A2 mistyped co-culture model.

**Methods and Results:**

Freshly isolated human PBMCs were co-cultured with human umbilical vein endothelial cells or human coronary arterial endothelial cells of converse human leukocyte antigen A2 (HLA-A2) status. This allows the tracking of the PBMC-derived cells by HLA-A2 expression and assessment of their phenotype pattern over time. PBMCs that adhered to the endothelium at the start of the co-culture were predominantly CD11b+ blood monocytes. After 24 to 72 hours in co-culture, the endothelium-adherent monocytes acquired endothelial-like properties including the expression of endothelial nitric oxide synthase, CD105, CD144 and vascular endothelial growth factor receptor 2. The expression of monocyte/macrophage lineage antigens CD14, CD11b and CD36 were down regulated concomitantly. The adherent monocytes did not express CD115 after 1 day of co-culture. By day 6, the monocyte-derived cells expressed vascular cell adhesion molecule 1 in response to tumour necrosis factor alpha. Up to 10% of the PBMCs adhered to the endothelium. These monocyte-derived cells contributed up to 30% of the co-cultured cell layer and this was dose-dependent on the PBMC seeding density.

**Conclusions:**

Human blood monocytes undergo rapid phenotype change to resemble endothelial cells after adhering to endothelium.

## Introduction

Monocytes, a subtype of the human peripheral blood mononuclear cell (PBMC) population, are key cells in the host immune system. However, recent evidence suggests an additional role of blood monocytes in stimulating endothelial proliferation [1] as well as acting as a potential source of endothelial-like cells [2], [3], [4]. Under physiological conditions, the interaction of circulating monocytes with the endothelial layer is critical to host defence and vascular health. To date, relatively little is known about the physical characteristics endothelium-adherent primary blood monocytes. In the studies reported in this paper, we utilized a novel HLA-A2 mistyped PBMC/endothelium co-culture model to assess phenotypic changes in human blood monocytes that adhere to endothelium.

It has previously been reported that the gene expression pattern in the human monocyte is radically altered after 2 hours of co-culture with endothelium [5]. Most importantly, the up-regulated genes in the endothelium-adherent monocytes include the endothelium-specific E-selectin and vascular cell adhesion molecule 1 (VCAM-1). One limitation of that study is the co-culture period was short (2 hours). The monocytes were identified by CD14 expression, which was utilized to select the monocytes out of the co-culture for gene expression analysis. Furthermore, changes in the expression of surface antigens on the adherent monocytes were not documented. The aim of the current study is to evaluate the phenotype change of endothelium-adherent monocyte in the longer term using a novel HLA-A2 mistyped PBMC/endothelium adherent co-culture model. PBMCs were obtained from either HLA-A2 positive (HLA-A2+) or HLA-A2 negative (HLA-A2−) donors and then co-cultured with human umbilical vein endothelial cells (HUVECs) or human coronary arterial endothelial cells (HCAECs) of the converse HLA-A2 status. This arrangement allowed the PBMC-derived cells to be tracked by their HLA-A2 expression without using surface antigens such as CD14, which may be altered during co-culture. HLA class I mistyping is an accepted method for discriminating and quantifying allogeneic cell populations in mixed leukocyte culture [6]. HLA-A2 mistyping has also been used to reliably track the *in vivo* fate of platelets after transfusion [7]. HLA-A2+ individuals represent 35–50% of the general population [8] and can be readily identified. This approach allows us to identify PBMC subtypes that adhered to the endothelium and track their subsequent phenotype change without having to label the cells with reagents that may interfere with their biology.

In this paper, we reported that the PBMC subtype that adhered to the endothelium is the CD11b+ monocyte and these adherent cells underwent a time-dependent phenotype change to resemble endothelial cells.

## Methods

### Ethics Statement

PBMCs were obtained from healthy human volunteers with written consent in accordance with the Declaration of Helsinki. This is classified as negligible risk to the donors and therefore do not require ethical approval according to section 5.1.22 of the Australian National Statement on Ethical Conduct in Human Research (2007).

### Preparation of the Endothelium

HUVECs were obtained from umbilical cords provided by the Royal Prince Alfred Hospital in Sydney. Each batch of HUVECs were isolated from a single umbilical cord using an established collagenase digestion technique [9] and was maintained in M199 culture medium (Invitrogen, Australia) supplemented with 10% foetal bovine serum (FBS, Invitrogen, Australia). HCAECs were obtained from Cell Applications, Inc (USA) and were maintained in Meso Endo Cell Growth Medium (Cell Applications, Inc. USA) before the start of the co-culture experiments. The HUVECs and HCAECs were screened for HLA-A2 status by immunofluorescence. Briefly, the cells were treated with trypsin, incubated for 45 min at 4°C with either an Alexa Fluor® 488 (AF488) conjugated mouse anti-human HLA-A2 monoclonal antibody (Serotec) or an AF488 conjugated mouse isotype control antibody (Serotec) and analysed with one-colour flow cytometry. HUVECs (passage 2, density from 0.1 to 0.3×10^6^ cells/well) were seeded onto 6 well (9.6 cm^2^/well) culture plates (Bectin Dickinson Labware) 24–48 h before the co-culture experiments.

### PBMC/Endothelium co-culture

Healthy human volunteers were screened for HLA-A2 status using direct immunofluorescence techniques as described above. Briefly, venous blood was collected from the donors into sterile EDTA-Na_2_ tubes, which was then diluted 1∶1 with phosphate buffered saline (PBS) and centrifuged at 200 g for 15 min to remove the platelet-rich supernatant. The PBMCs were isolated by density gradient centrifugation (Lymphoprep™) and washed in PBS at 1∶1 dilution. The PBMCs were incubated with either an AF488 conjugated mouse anti-human HLA-A2 monoclonal antibody (Serotec) or an AF488 conjugated mouse isotype control antibody (Serotec) and analysed with one-colour flow cytometry.

For the co-culture experiments, HLA-A2+ PBMCs were added immediately after isolation at densities ranging from 0.25 to 2×10^6^ cells/well onto HLA-A2− HUVEC layers. Reverse HLA-A2 mistyping co-culture experiments with HLA-A2− PBMCs and HLA-A2+ HUVECs were also performed. The PBMCs and HUVECs were co-incubated at 37°C for 2 h in M199 supplemented with either 10% FBS or heat-inactivated human serum (HIHS), after which the non-adherent PBMCs were removed by washing. The cell layers were analysed by three-colour flow cytometry at serial time points between day 0 and day 6 of co-culture. For each experiment, PBMCs from a single donor were co-cultured with HUVECs from a single source and the medium was supplemented with a single batch of serum. To assess VCAM-1 expression, the co-cultures were stimulated for 24 h with TNF-α (10 ng/ml) on day 5 of co-culture. Co-culture experiments were also done with PBMCs and HCAECs using the same techniques and under the same culture conditions for comparison.

### Immunofluorescence and flow cytometry

Three-colour direct immunofluorescence flow cytometry (Cytomics FC500, Beckman Coulter) was used to characterize and quantify the HLA-A2+ cells. The co-culture cell layers were dissociated with either trypsin or by gentle scraping. Total cell counts were estimated using a manual haemocytometer. For detection of surface antigens, the detached cells were incubated for 45 minutes at 4°C with AF488, R-phycoerythrin (R-PE) and PE-Cy5 conjugated primary antibodies (1∶50 or 1∶100). For the detection of intracellular antigens, the cells were fixed and permeabilized using the Fix & Perm® kit (Invitrogen). The AF488 conjugated monoclonal mouse anti-human HLA-A2 antibody (Serotec) was used to detect HLA-A2 expression. R-PE-conjugated monoclonal antibodies included mouse anti-human CD31 (BD Biosciences), eNOS (BD Biosciences), CD105 (Serotec), CD144 (Santa Cruz and R&D Systems), CD14 (BD Biosciences), CD11b (BD Biosciences), CD16 (BD Biosciences), CD36 (BD Biosciences), CD115 (R&D Systems), VEGFR2 (R&D Systems), CD34 (BD Biosciences), ICAM-1 (BD Biosciences) and VCAM-1 (BD Biosciences). The PE-Cy5-conjugated CD11b (BD Biosciences) antibody was used for 3-colour flow cytometry analysis. AF488, R-PE and PE-Cy5 conjugated mouse isotype control antibodies as recommended by the manufacturers were used to exclude non-specific binding. HUVECs that had not been subjected to co-culture with PBMCs were incubated with the HLA-A2 AF488 antibody as cell controls. Fluorescence images were acquired with a Olympus IX71 microscope using the manufacturer's software.

### Statistical analysis

Data sets were compared with a two-tailed unpaired Student's t-test (Microsoft Excel) and P<0.05 was considered statistically significant.

## Results

### Effects of co-culture duration on the phenotype of endothelial-adherent monocytes

**Figure 1:**
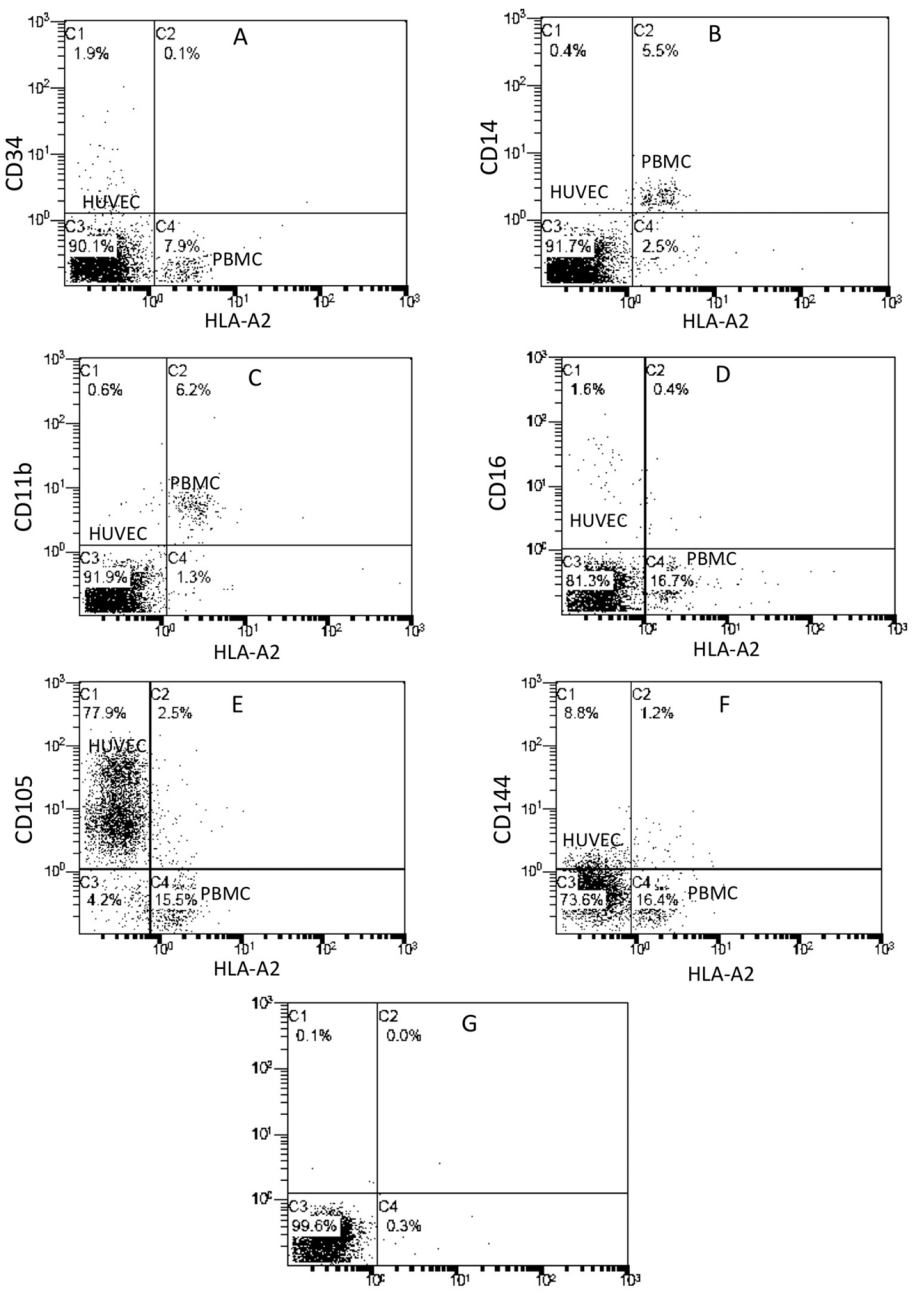
Endothelial adherence of blood monocytes. PBMCs were isolated from HLA-A2+ donors and incubated with HLA-A2− HUVECs (1×10^6^ cells/well) for 2 h, after which the non-adherent PBMCs were removed by washing. The co-cultured cell layers were immediately analysed with dual-colour flow cytometry for HLA-A2 and (A) CD34, (B) CD14, (C) CD11b, (D) CD16, (E) CD105 and (F) CD144 expression. Representative plots from 4–6 individual experiments are shown. (G) Two parameters dot plot showing typical isotype controls.

After 2 h of co-incubating HLA-A2+ PBMCs with HLA-A2− HUVECs, the endothelium-adherent HLA-A2+ cells were CD34−/CD14+/CD11b+/CD16− (Figure 1A to D) and were predominantly CD105−/CD144− (Figure 1E and F). Thus the phenotype of the endothelium-adherent PBMCs was consistent with that of blood monocytes.

**Figure 2:**
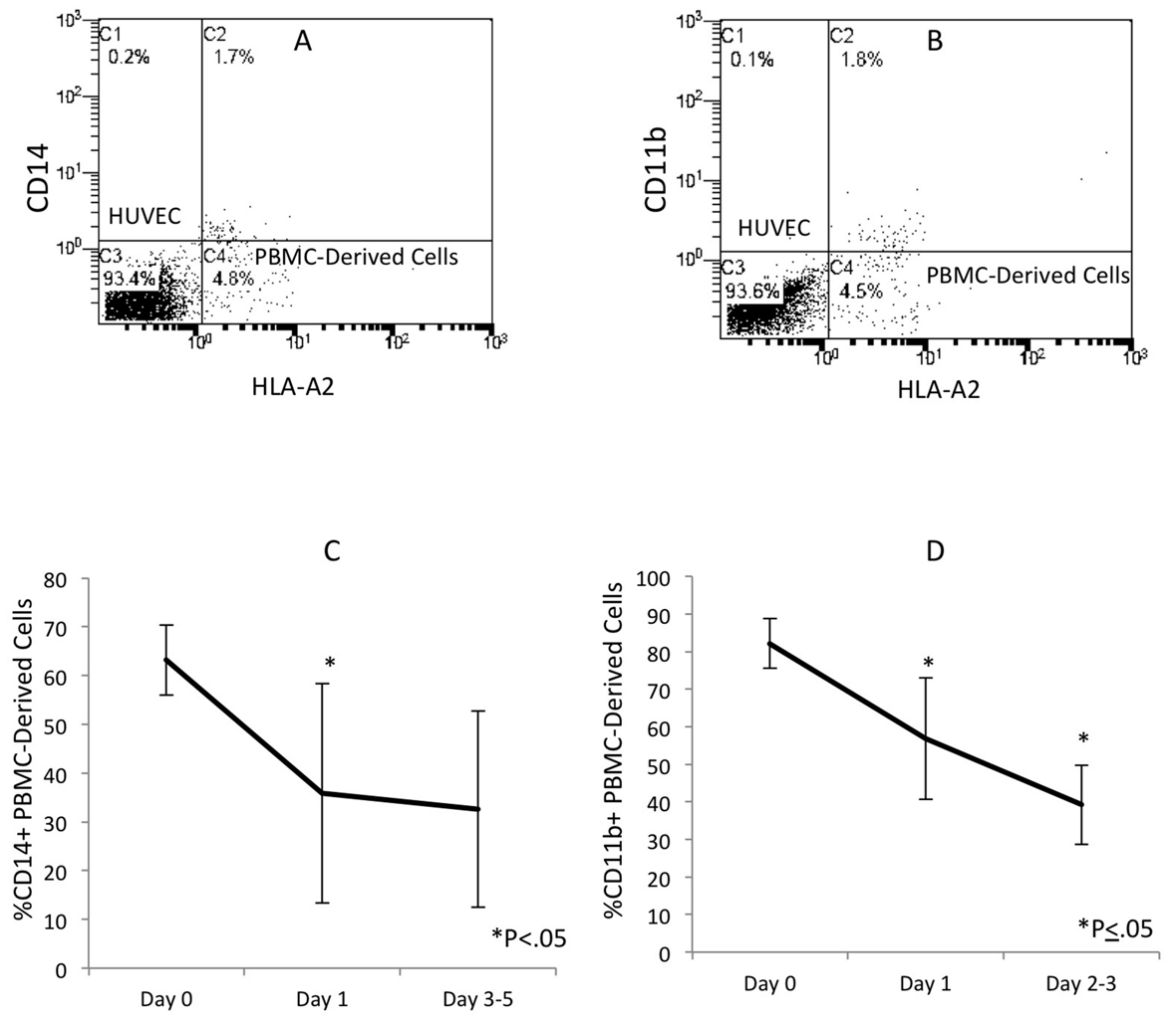
Down-regulation of CD14 and CD11b expression in the endothelium-adherent monocytes during co-culture. HLA-A2+ PBMCs (1×10^6^ cells/well) were incubated for 2 h (Day 0) with HLA-A2− HUVECs, after which the non-adherent cells were removed by washing. The cell layers were assessed by dual-colour flow cytometry for HLA-A2 and (A) CD14 expression on Day 1 and (B) CD11b expression on Day 3 of co-culture. These are representative plots from 4–7 individual experiments. The reduction in CD14 expression from Day 0 to Day 5 (C) and CD11b expression from Day 0 to Day 3 (D) is also shown.

After 24 h of co-culture, the expression of CD14 and CD11b on the HLA-A2+ adherent monocytes was markedly down-regulated (Figures 2A and B). At the 2 h time point, 63.2±6.6% of the HLA-A2+ cells expressed CD14; after 24 h of co-culture only 35.8±22.5% of the HLA-A2+ cells expressed CD14 (P = 0.02). The percentage of the HLA-A2+ cells that expressed CD14 remained at 32.6±20.1 for up to 5 days of co-culturing (Figure 2C). Similarly, 82.2±6.6% of the HLA-A2+ cells expressed CD11b at the 2 h time point but this was reduced to 56.9±16.2% by 24 h (P<0.01). After 2 to 3 days of co-culture, the percentage of HLA-A2+ cells that expressed CD11b was further reduced to 39.2±10.5% (P = 0.05 compared to 24 h) (Figure 2D).

**Figure 3:**
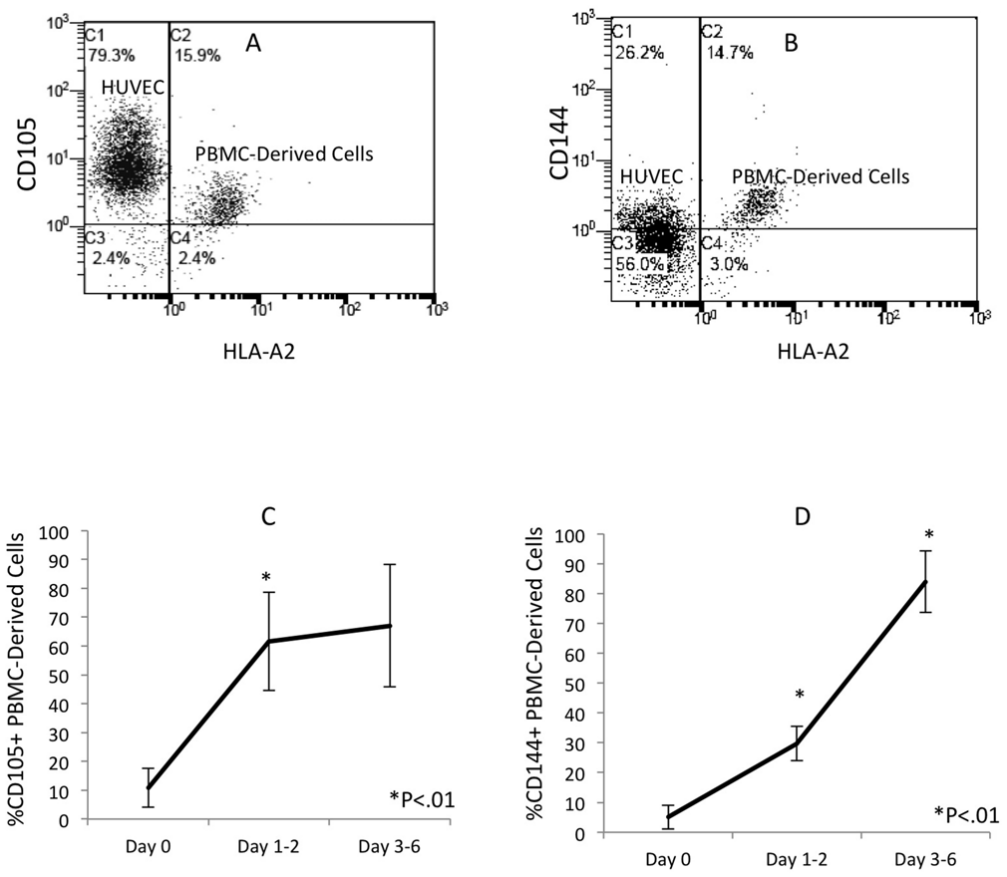
Increased expression of CD105 and CD144 in the endothelium-adherent monocytes during co-culture. HLA-A2+ PBMCs (1×10^6^ cells/well) were incubated for 2 h (Day 0) with HLA-A2− HUVECs, after which the non-adherent cells were removed by washing. The cell layers were maintained in co-culture up to Day 6, then assessed by dual-colour flow cytometry for HLA-A2 and (A) CD105 and (B) CD144 expression on Day 3 of co-culture. Representative plots from 4–7 individual experiments are shown. The increase in CD105 from Day 0 to Day 6 (C) and CD144 expression from Day 0 to Day 6 (D) is also shown.

**Figure 4:**
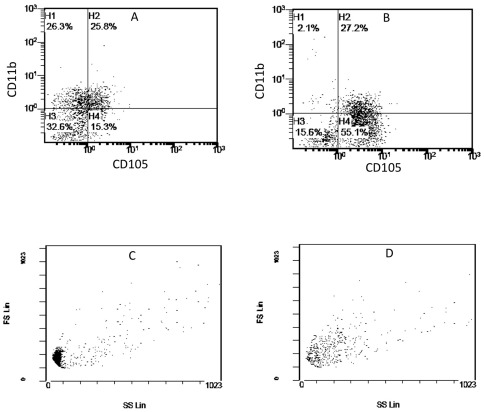
Phenotype change from HLA-A2+/CD11b+/CD105− to HLA-A2+/CD11b−/CD105+ on endothelium-adherent blood monocyte-derived cells with increase in size and granularity during co-culture. HLA-A2+ PBMCs (1×10^6^ cells/well) were incubated for 2 h (Day 0) with HLA-A2− HUVECs, after which the non-adherent cells were removed by washing. The cell layers were analysed by three-colour flow cytometry staining for HLA-A2, CD11b and CD105 on (A) Day 1 and (B) Day 2. These plots were gated for HLA-A2+ cells. Forward scatter/side scatter dot plots gated for HLA-A2+ cells on Day 0 (C) and Day 2 (D) was shown. These are representative of 2 individual experiments.

Coinciding with this down-regulation of monocyte antigens, the endothelium-adherent HLA-A2+ monocytes began to express the endothelial antigens CD105 (Figure 3A) and CD144 (Figure 3B). At 2 h, only 10.8±6.7% of the HLA-A2+ cells expressed CD105 (Figure 3C). By 24–48 h, 61.6±17% of the HLA-A2+ cells expressed CD105 (P<0.01). This value remained constant up to Day 6 of co-culture (Figure 3C). At the 2 h time point, only 5.1±4% of the HLA-A2+ cells expressed CD144 (Figure 3D), while after 24–48 h, 29.7±5.8% of these cells expressed CD144 (P<0.01). After 3 to 6 days in co-culture, CD144 expression by the HLA-A2+ cells was further increased to 83.9±10.3% (P<0.01 compared to the 24–48 h time point) (Figure 3D). Three-colour flow cytometry analysis of the simultaneous staining for HLA-A2, CD105 and CD11b showed a clear shift of the HLA-A2+/CD11b+/CD105− cells into HLA-A2+/CD11b−/CD105+ cells between day 1 (Figure 4A) and day 2 (Figure 4B) of co-culture. This is consistent with simultaneous down regulation of CD11b and up regulation of CD105 within the same cell population during the 24 hour period.

In addition to the changes in the endothelial and monocytic antigens, the monocyte-derived cells increased in size and granularity from 2 h (Figure 4C) to day 2 (Figure 4D) of co-culture as demonstrated by the forward scatter/side scatter dot plots.

The adherent monocytes did not express the M-CSF receptor CD115 after 1 day in co-culture and remained CD34− after 2 days in co-culture (data not shown).

To exclude the possibility that the HLA-A2 antigen may be transferred from microparticles derived from contaminating HLA-A2+ platelet or monocytes to the HLA-A2− HUVECs, the HLA-A2 mistyping was reversed (HLA-A2− PBMCs with HLA-A2+ HUVECs) and analysed on day 2 of co-culture. This confirmed that 33.7±8.9% of the HLA-A2− adherent monocytes expressed CD105 and that expression of CD14 (9.4±1.2% of the adherent monocytes) and CD11b (18.4±8.6% of the adherent monocytes) was low.

### Expression of VEGFR2, adhesion molecules and CD36 by endothelium-adherent monocytes

**Figure 5:**
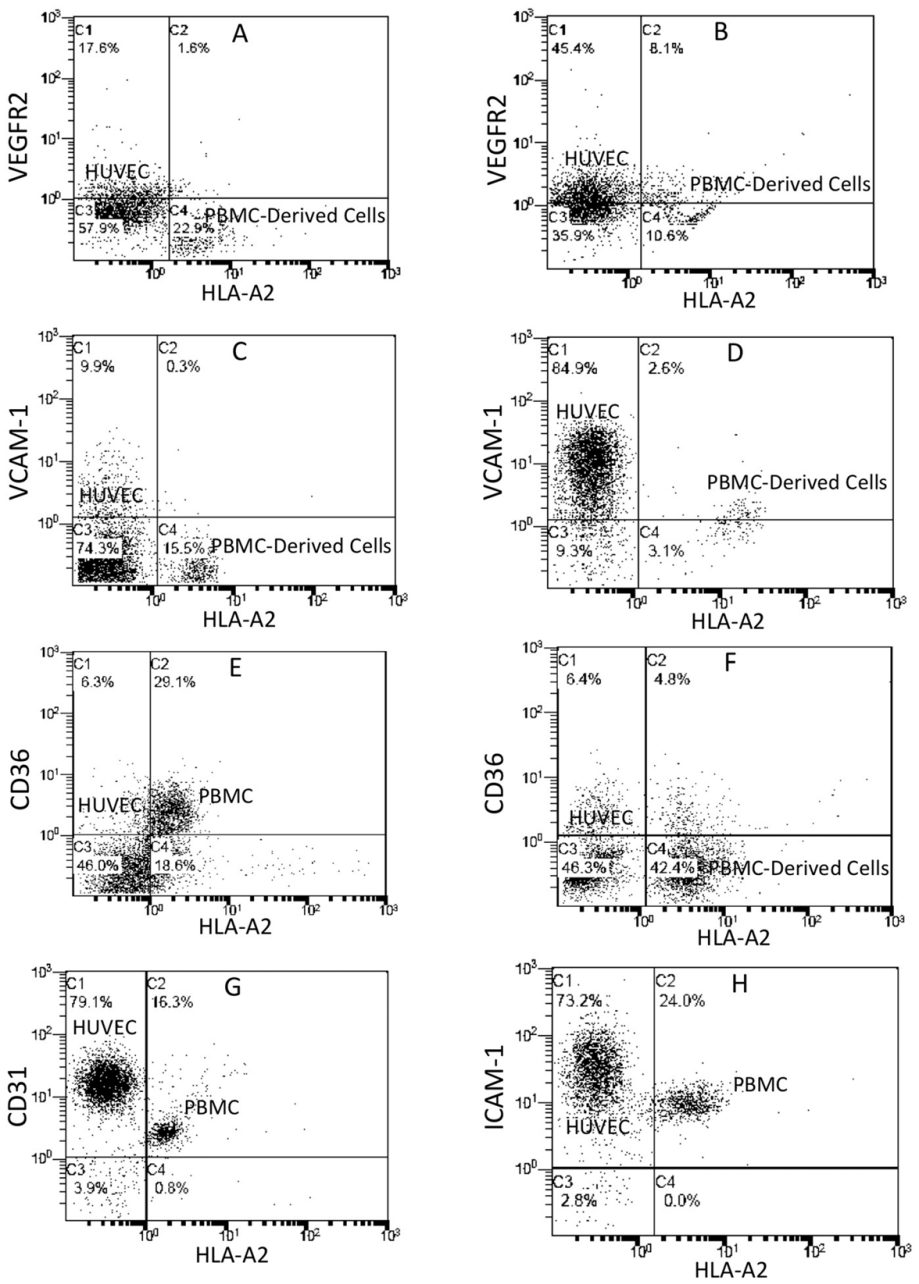
Increased expression of VEGFR2 and VCAM-1, reduced expression of CD36 and expression of CD31 and ICAM-1 in the endothelium-adherent monocytes in co-culture. HLA-A2+ PBMCs (1×10^6^ cells/well) were incubated for 2 h (Day 0) with HLA-A2− HUVEC, after which the non-adherent cells were removed by washing. The cell layers were analysed by dual-colour flow cytometry for HLA-A2 and VEGFR2 expression on Day 1 (A) and Day 2 (B) of co-culture, VCAM-1 expression in the absence of TNF-α on Day 6 of co-culture (C), VCAM-1 expression on Day 6 of co-culture after 24 hours of stimulation with TNF-α (10 ng/ml) (D), CD36 expression on Day 0 (E) and Day 1 (F) of co-culture, as well as CD31 (G) and ICAM-1 (H) expression on Day 0. Representative plots from 2–5 individual experiments are shown.

To determine whether the phenotype changes to the endothelium-adherent monocytes were accompanied by expression of molecules that are required for endothelial function, VEGFR2, endothelial cell adhesion molecules and the class B scavenger receptor, CD36 levels were quantified. After 24 h of co-culture, the HLA-A2+ adherent monocytes were still predominantly VEGFR2- (Figure 5A). However, by 48 h, 43.3% of the adherent monocytes expressed VEGFR2 (Figure 5B). In the absence of stimulation by TNF-α, VCAM-1 was not expressed by the HLA-A2+ monocytes either at 2 h (result not shown) or at Day 5 of co-culture (Figure 5C). After 5 days of co-culture, activation with TNF-α induced VCAM-1 expression in 45.6% of the HLA-A2+ cells (Figure 5D). At 2 h, CD36 was expressed by the majority of the endothelium-adherent monocytes (Figure 5E), but after 24 h, expression of this receptor by the adherent monocytes was reduced to only 10% (Figure 5F). All the endothelial-adherent HLA-A2+ cells expressed CD31 (Figure 5G) and ICAM-1 (Figure 5H) at 2 h of co-culture consistent with constitutive expression of these antigens. This was maintained for up to 4 days in co-culture (result not shown).

### Endothelial nitric oxide synthase expression

**Figure 6:**
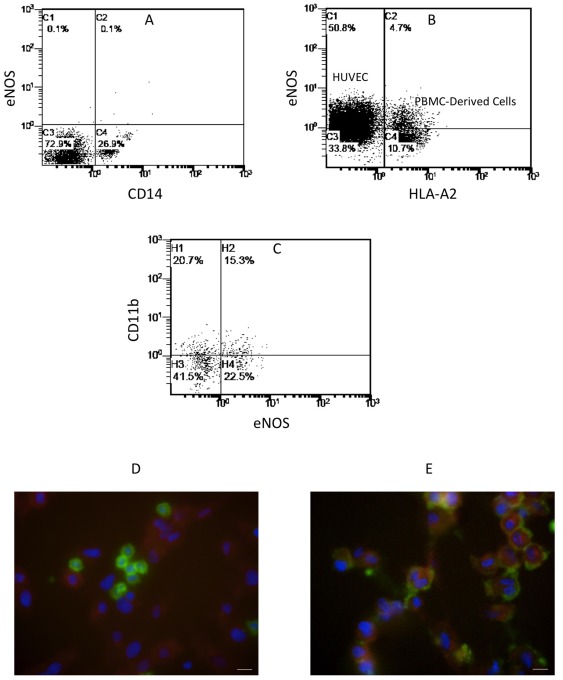
Expression of eNOS in endothelium-adherent monocytes in co-culture. Dual-colour flow cytometry analysis of CD14 and eNOS expression on freshly-isolated PBMCs before co-culture (representative of 2 individual experiments) is shown (A). HLA-A2+ PBMCs (1×10^6^ cells/well) were incubated for 2 h with HLA-A2− HUVECs, after which the non-adherent PBMCs were removed by washing. (B) Shows the flow cytometry analysis of the cell layers for HLA-A2 and eNOS expression on Day 1 of co-culture (representative of 3 individual experiments). (C) Shows the 2 parameters dot plot from 3-colour flow cytometry analysis on Day 1 of co-culture staining for HLA-A2, CD11b and eNOS. This plot is gated for HLA-A2+ cells (representative of 2 individual experiments). Immunofluorescent micrographs (×60 original magnification) showing endothelial-adherent HLA-A2+ cells (green) and eNOS expression (red) on Day 0 (D) and Day 1 (E) of co-culture (representative of 2 individual experiments). The nuclei were stained with DAPI. Scale bar = 20 µm.

We also investigated the expression of endothelial nitric oxide synthase (eNOS) by the endothelium-adherent monocytes in co-culture, which is a critical indicator of endothelial functionality. Freshly isolated PBMCs did not express eNOS prior to co-culture (Figure 6A). In contrast, 30.5% of the HLA-A2+ cells expressed eNOS after 24 h in co-culture (Figure 6B). Three-colour flow cytometry with simultaneous staining for HLA-A2, CD11b and eNOS showed that the majority of the HLA-A2+/eNOS+ cells were CD11b− (Figure 6C). Expression of eNOS was visualized with fluorescence microscopy after 2 h and 24 h of co-culture. At 2 h, the adherent HLA-A2+ cells (green) were small round cells that were eNOS− (Figure 6D). After 24 h, these cells increased in size and became eNOS+ (red, Figure 6E), consistent with the flow cytometry analysis.

Collectively, these phenotype changes to the HLA-A2+ cells in combination with the increase in size and granularity provide compelling evidence that the endothelial-adherent monocytes acquired endothelial-like properties and we termed this monocyte-derived endothelial-like cells (M-ELCs).

The use of either FBS or HIHS to enrich the culture medium had no effect on the phenotype changes of these adherent monocytes (result not shown).

### PBMC/HCAEC Co-Culture

**Figure 7:**
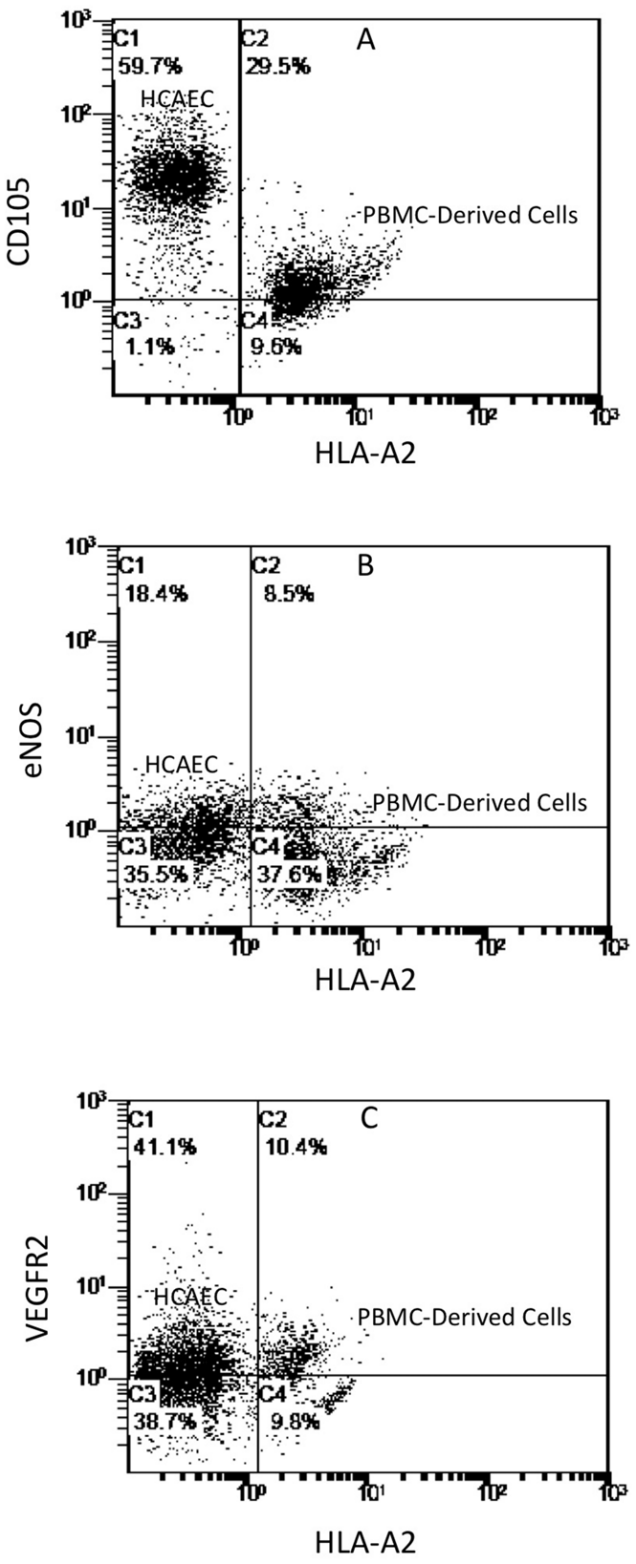
Expression of endothelial antigens in endothelium-adherent monocytes in co-culture with HCAECs. HLA-A2+ PBMCs (1×10^6^ cells/well) were incubated for 2 h (Day 0) with HLA-A2− HCAECs, after which the non-adherent cells were removed by washing. The cell layers were analysed by dual-colour flow cytometry for HLA-A2 and (A) CD105, (B) eNOS and (C) VEGFR2 expression on Day 2 of co-culture.

To assess whether the changes of the blood monocytes in co-culture with endothelium are specific to HUVECs, further co-culture experiments were done using HCAECs. The phenotype of the PBMCs that adhered to the HCAEC by 2 h of co-culture was identical to that in the PBMC/HUVEC co-cultures (data not shown). The phenotype changes by Day 2 of co-culture were also identical to the PBMC/HUVEC co-cultures with acquisition of CD105 (Figure 7A), eNOS (Figure 7B) and VEGFR2 (Figure 7C). CD14 and CD11b were down regulated (data not shown). Hence, the phenotype changes of the endothelium-adherent blood monocytes were not specific to the use of HUVECs.

### Quantification of M-ELC generation

When HLA-A2+ PBMCs were seeded at a dose of 1–1.5×10^6^ cells/well onto confluent HLA-A2− HUVEC layers, 9–30% of the total co-culture cell layer was HLA-A2+ after 2 h of co-culture. The number of PBMCs that adhered to the HUVECs represented 4–10% of the PBMC population that was initially seeded. The percentage of HLA-A2+ cells in the co-culture cell population remained constant for up to 3 days.

**Figure 8:**
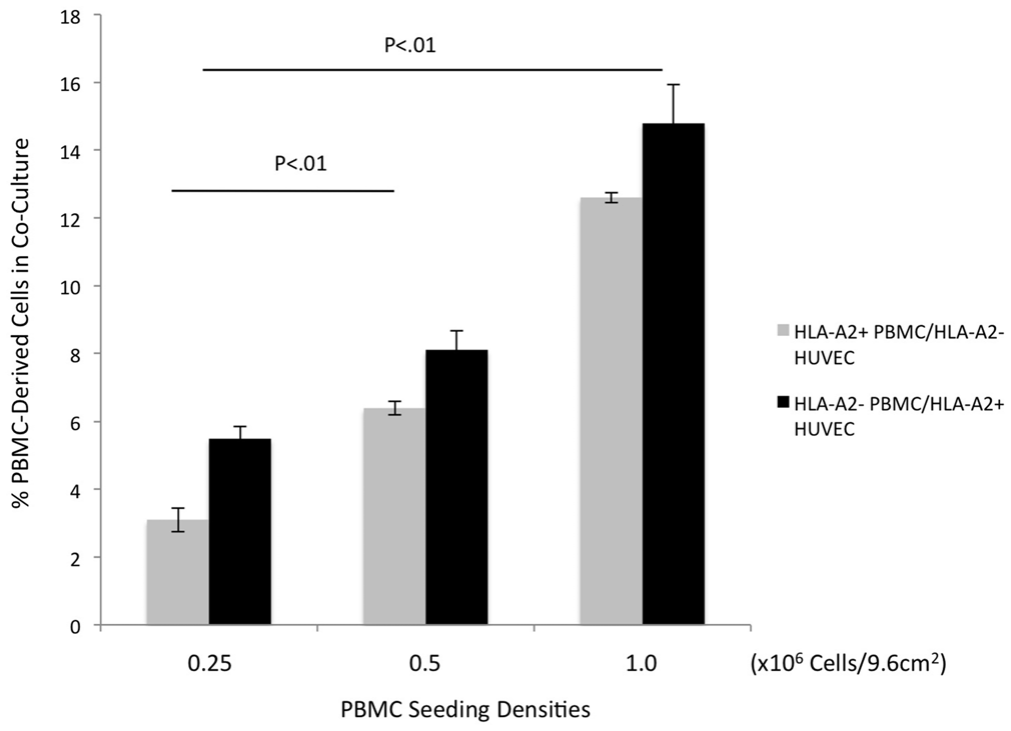
Quantification of endothelium-adherent monocytes in co-culture. PBMCs were seeded onto HUVECs of converse HLA-A2 status, co-incubated for 2 h, after which the non-adherent cells were washed off. The percentage of HLA-A2+ cells was evaluated using dual-colour flow cytometry after 24–48 h of co-culture. The graph shows the effect of varying the PBMC seeding densities at 0.25×10^6^, 0.5×10^6^ and 1.0×10^6^ cells/well. The % HLA-A2+ PBMCs co-cultured with the HLA-A2− HUVECs (grey bars) and the % HLA-A2− PBMCs co-cultured with HLA-A2+ HUVECs (black bars) are shown.

The effect of varying the seeding density of PBMCs on the percentages of the M-ELCs in the total co-culture population was evaluated using low (0.25×10^6^), intermediate (0.5×10^6^) and high (1×10^6^) numbers of PBMCs/well. For any given experiment described in this section, a single batch of HLA-A2− HUVECs was seeded at a density of 0.15×10^6^ cells/well. The HLA-A2+ PBMCs were taken from a single donor. Non-adherent PBMCs were removed by washing after 2 h and the incubation continued for 24 h. When added at a seeding density of 0.25×10^6^ cells/well, HLA-A2+ cells accounted for 3.1±0.4% of the total cells in the co-culture layer after 24 h of co-culture. The proportion of HLA-2+ cells in the co-culture layer after 24 h of incubation increased to 6.3±0.2% when seeded at a density of 0.5×10^6^ PBMCs/well and increased further to 12.6±0.2% when seeded at a density of 1×10^6^ PBMCs/well (Figure 8). These percentages and the total cell counts were used to establish that the low, intermediate and high dose PBMC groups contained 20,000, 50,000 and 150,000 HLA-A2+ cells per well, respectively. Thus, the number of M-ELC after 24 h of culture was proportional to the PBMC seeding density and represented 7.6% to 15% of the starting PBMC population. Reversing the HLA-A2 mistyping with HLA-A2− PBMC and HLA-A2+ HUVEC revealed a similar PBMC dose-response, with 5.5±0.4% M-ELCs in the 0.25×10^6^ PBMC/well group, 8.1±0.6% in the 0.5×10^6^ PBMC/well group and 14.8±1.2% in the 1.0×10^6^ PBMC/well group (Figure 8).

The effect of varying the seeding density of the HUVECs on the proportion of M-ELC was evaluated by plating the HLA-A2− HUVECs at low (0.1×10^6^ cells/well), intermediated (0.2×10^6^ cells/well) and high (0.3×10^6^ cells/well) density 24 h before adding the HLA-A2+ PBMCs. The PBMCs were added to the HUVECs at 1×10^6^ cells/well in all groups. Non-adherent PBMCs were washed off after 2 h and the co-culture was maintained for 2 days. Under these conditions the percentage of HLA-A2+ cells in the low, medium and high HUVECs seeding densities remained remarkably stable at 14.7±1.3%. The total co-cultured cell counts were 0.17×10^6^, 0.35×10^6^ and 0.57×10^6^ cells/well in the low, medium and high HUVEC seeding densities, respectively. This finding suggests that the percentage of M-ELCs in the co-culture is independent of HUVEC density.

## Discussion

The key finding in this study is that the majority of human blood monocytes that adhered to the endothelial layer underwent time-dependent change into an endothelial-like phenotype that is distinct from the typical monocyte/macrophage lineage.

Human blood monocytes express endothelial antigens under angiogenic conditions [2], [3], [4] and blood monocytes in direct contact with endothelium express endothelium-specific genes [5]. Furthermore, human monocytes/macrophages cultured for 10–14 days *ex vivo* are known to acquire endothelial-like morphology and also expressed endothelial-specific antigens when added to HUVEC after a further culture period of 7 days [10]. The current study is the first to show that when primary blood monocytes adhere to endothelial cells, they change from a typical monocyte phenotype to a phenotype resembling that of endothelial cells.

Antigens expressed by endothelium-adherent monocytes differed markedly from that of typical blood monocytes. In contrast to typical blood monocytes, endothelium-adherent monocytes expressed a number of endothelial antigens, including CD105, CD144, VEGFR2, VCAM-1 and eNOS. CD105 is a component of the transforming growth factor-beta receptor system that is typically expressed by activated endothelium [11]. CD144 (VE-cadherin) is a component of the highly specific endothelial adherent junction [12], [13], [14]. VEGFR2 is a receptor of vascular endothelial growth factor that is specific to endothelium. Furthermore, fresh, non-adherent monocytes do not express VCAM-1, either in the basal or activated state. In the current co-culture model, we found that activation by TNF-α induced VCAM-1 expression in the endothelium-adherent monocytes, which is a well-documented feature of endothelial cells [15]. In addition, the adherent (but not fresh) monocytes expressed eNOS, an intracellular enzyme that plays a key role in regulating the vasomotor tone that defines endothelial functionality.

Expression of the monocyte integrin CD11b is normally increased in monocytes during cell activation and macrophage differentiation [16], [17], [18]. However, in the endothelium-adherent monocytes, CD11b was down regulated, providing further evidence of a shift away from the monocyte/macrophage lineage. The scavenger receptor CD36 is another monocyte/phagocyte antigen [19] that contributes to the uptake of modified LDLs and is fundamental for monocyte-macrophage differentiation [20], [21]. We found that CD36 expression in endothelium-adherent monocytes was down regulated after 24 hours of co-culture, providing yet another indication that these cells had shifted away from their phagocytic lineage. Collectively, the acquisition of endothelial antigens combining with the loss of monocytic/phagocytic antigens strongly indicated that the endothelium-adherent monocytes had differentiated into cells with the phenotype and functionality of typical endothelial cells. The current findings highlight the potential of blood monocyte as a substantial source of endothelial cell replacement.

Platelet or monocyte-derived microparticles are unlikely to be involved in this process as the reverse HLA-A2 mistyping exclude the transfer of HLA-A2 via microparticles. Furthermore, platelet microparticles promote macrophage differentiation of monocytes [22], which is opposite to our observations. The mechanism by which contact with the endothelium results in the differentiation of blood monocytes into endothelial-like cells remains to be determined.

In conclusion, a substantial proportion of circulating monocytes has the potential to rapidly transform into endothelial-like cells after adhering to pre-existing endothelium. This process may be beneficial in promoting vascular repair and improving vascular health.
